# Tumor Microenvironment and Immune Escape in the Time Course of Glioblastoma

**DOI:** 10.1007/s12035-022-02996-z

**Published:** 2022-09-01

**Authors:** Assunta Virtuoso, Ciro De Luca, Giovanni Cirillo, Matteo Riva, Gabriele Romano, Angela Bentivegna, Marialuisa Lavitrano, Michele Papa, Roberto Giovannoni

**Affiliations:** 1 Laboratory of Neuronal Networks Morphology and System Biology, Department of Mental and Physical Health and Preventive Medicine, University of Campania “Luigi Vanvitelli”, 80138 Naples, Italy; 2 School of Medicine and Surgery, University of Milano-Bicocca, 20900 Monza, Italy; 3 Laboratory of Tumor Immunology and Immunotherapy, Department of Oncology, Leuven Cancer Institute, KU Leuven, 3000 Leuven, Belgium; 4 Department of Neurosurgery, CHU UCL Namur, University Hospital of Godinne, 5530 Yvoir, Belgium; 5 Department of Pharmacology & Physiology, College of Medicine, Drexel University, 245 North 15th Street, NCB 8814A, Philadelphia, PA 19102 USA; 6 SYSBIO Centre of Systems Biology ISBE-IT, Milan, Italy; 7 Dept of Biology, University of Pisa, 56126 Pisa, Italy

**Keywords:** Microglia, Macrophages, Astrocytes, Spatio-temporal heterogeneity, Neuroinflammation, Glioma, MMP-9, MHCII, FIB-2

## Abstract

Glioblastoma multiforme (GBM) is the most aggressive primary brain tumor with a malignant prognosis. GBM is characterized by high cellular heterogeneity and its progression relies on the interaction with the central nervous system, which ensures the immune-escape and tumor promotion. This interplay induces metabolic, (epi)-genetic and molecular rewiring in both domains. In the present study, we aim to characterize the time-related changes in the GBM landscape, using a syngeneic mouse model of primary GBM. GL261 glioma cells were injected in the right striatum of immuno-competent C57Bl/6 mice and animals were sacrificed after 7, 14, and 21 days (7D, 14D, 21D). The tumor development was assessed through 3D tomographic imaging and brains were processed for immunohistochemistry, immunofluorescence, and western blotting. A human transcriptomic database was inquired to support the translational value of the experimental data. Our results showed the dynamic of the tumor progression, being established as a bulk at 14D and surrounded by a dense scar of reactive astrocytes. The GBM growth was paralleled by the impairment in the microglial/macrophagic recruitment and antigen-presenting functions, while the invasive phase was characterized by changes in the extracellular matrix, as shown by the analysis of tenascin C and metalloproteinase-9. The present study emphasizes the role of the molecular changes in the microenvironment during the GBM progression, fostering the development of novel multi-targeted, time-dependent therapies in an experimental model similar to the human disease.

**Supplementary Information:**

The online version contains supplementary material available at 10.1007/s12035-022-02996-z.

## Background

Glioblastoma multiforme (GBM) is the most frequent and aggressive primary brain tumor with a malignant prognosis. The success of standard therapies is hampered by the neoplastic cell heterogeneity and the high aggressiveness of the tumor. Surgical resection is hindered by tumor location and early central nervous system (CNS) invasion [1]. Probably due to its glial nature [2], GBM is integrated within the CNS tissue and is incapable of forming extra-CNS metastases. GBM needs resident brain cells for survival and progression in a mutually forged unique microenvironment. The GBM microenvironment is dynamic and maintained by a metabolism-based equilibrium between neoplastic cells and resident cells, alternating hypoxic niches, pseudopalisades, proliferating areas, microvascular angiogenesis, and migration-permissive zones [3]. As the innate immune system, microglia, astrocytes, and macrophages are the first cells to potentially counteract the tumor. Activated microglia, macrophages, and astrocytes should protect the nervous system and maintain homeostasis. However, the resident microglia and marginating macrophages could acquire a tumor-supportive behavior, probably due to GBM-induced signaling [4]. Microglia/macrophages are recruited by GBM release of CCL-2 (macrophage chemoattractant protein-1) to promote the tumor invasiveness [5, 6]. Tumor cells are heterogeneous and could contribute to immune system silencing/activation, and extracellular matrix (ECM) modifications to favor tumoral immune escape, growth, and progression. Among the ECM components, matrix metalloproteases (MMPs), particularly MMP-9, are involved in tissue remodeling and GBM invasiveness. Tenascin-C (TN-C) is an ECM glycoprotein that correlates with the GBM behavior *to grow or to go* [7]. Fibulin-2 is a large calcium-binding ECM with a central role in both physiological integrity of the CNS and carcinogenesis [8]. Fibulin-2 role in GBM progression has not been established yet, but it was found associated with advanced clinical stages in II/III/IV grade astrocytomas [9].

Recent data suggest a combined approach for GBM therapy: directly fighting the tumor and conditioning the engagement of surrounding healthy cells [3, 10, 11]. Resident cells polarization is central in the definition of a hostile microenvironment for tumor development. However, the intrinsic plasticity of tumor-CNS interactions leads to a complex pathophysiology continuously evolving. In the present study, we characterized the time-related changes in the tumor microenvironment (TME), using a syngeneic mouse model of primary GBM. We assessed the tumor development and we found a spatiotemporal heterogeneity of glial cells, innate immune system, and ECM response during the GBM progression, based on the cell type, disease phases, and anatomical distribution.

## Material and Methods

### Animals

Female adult (4–6 week old) C57Bl/6 mice (Envigo) (*n* = 37) were included in the present study. Animal care was in compliance with the Italian and European Guidelines for use and care of laboratory animals (EU Directive 2010/63) under the authorization no. 268/2012-B from the Minister of Health. Each animal was allowed free access to food and water, under a 12/12 h light/dark cycle.

### Stereotactic Intracerebral Tumor Cell Inoculation

Mice were anesthetized via intraperitoneal (IP) injection of 6 µl/g body weight of a mixture of 18.75 mg/ml ketamine (Pfizer) and 0.125% xylazine (Bayer). GL261 glioma cells were obtained from Dr. Ilker Eyupoglu (University of Erlangen, Germany). 5 × 10^3^ GL261 cells were injected intracranially in C57Bl/6 mice following a procedure adapted from a previous publication [12] by means of a stereotactic frame (day 0). The cells were suspended in 4 µl of DMEM/F-12 and inoculated in the right striatum, 2 mm lateral from the midline, 2 mm anterior to the bregma, and 3 mm below the dura mater with a 26-gauge syringe (Hamilton). Animals belonging to the control group (*n* = 10) were inoculated with vehicle solution (DMEM/F-12) and will be mentioned in the text as the SHAM group. Afterward, the mice were weighed and clinical symptoms were evaluated at least three times per week. Mice were euthanized at 7, 14, or 21 days after the cells injection (7D, 14D, 21D experimental groups, respectively) or when they (n=2) lost 20% of their initial weight and reached grade 3–4 symptoms on a scoring system described in [13].

### In-Vivo 3D Fluorescence Tomography

Mice belonging to the SHAM (*n* = 1) and 21D (*n* = 4) groups were intravenously injected with 10 nmoles of Xenolight Rediject 2-DG-750 probe (Perkin Elmer) for non-invasive, in-vivo targeting of tumor, and imaged at 3 h post-injection with FMT 1000 Imaging System (Perkin Elmer). During the scan time, the mice were kept under isoflurane anesthesia. Scan images were then processed and 3D-elaborated using True Quant software (Perkin Elmer) for data management, automatic reconstruction, and high throughput data analysis.

### Tissue Preparation

Mice were deeply anesthetized on day 7 (7D Tumor *n* = 9; 7D SHAM n  = 4), 14 (14D Tumor *n* = 9; 14D SHAM *n* = 3) or 21 (21D Tumor *n* = 7; 21D SHAM *n* = 3) after tumor inoculation with an i.p. injection of 50 mg/kg Pentobarbital (Lundbeck) and transcardially perfused with saline solution (TrisHCl 0.1 M/ EDTA 10 mM). Fresh brains (*n* = 13) were immediately frozen by immersion in liquid nitrogen and stored at – 80 °C until protein extraction and western blotting analyses. Brains for immunostaining and histology (*n* = 22) continued with fixation by 4% paraformaldehyde in 0.01 M phosphate-buffered saline (PBS), pH 7.4 at 4 °C, before extraction. They were post-fixed O/N in the same fixative, then soaked in 30% sucrose PBS, and frozen in chilled isopentane on dry ice. Serial sections were cut at the slide microtome (25 μm thickness) and collected in cold PBS.

### Histology and Immunohistochemistry

Brain sections were blocked in 10% normal serum in 0.01 M PBS containing 15mM NaN3 and 0.25% Triton for 1 h at room temperature (RT). Each primary antibody was diluted in the blocking solution. Following 48-h incubation at 4 °C, sections were washed six times (10 min each) in PBS and incubated with the appropriate biotinylated secondary antibody (Vector Labs Inc., Burlingame, CA, USA; 1:200) for 90 min at RT, washed in PBS, and processed using the Vectastain avidin–biotin-peroxidase kit (Vector Labs Inc., Burlingame, CA, USA) for 90 min, at RT. Sections were washed in 0.05 M Tris–HCl and reacted with 3,3-diaminobenzidine tetrahydrochloride (DAB; Sigma, 0.5 mg/ml in Tris–HCl) and 0.01% hydrogen peroxide. Floating sections were mounted on chrome-alum gelatine-coated slides, dehydrated, and coverslipped. After mounting, serial sections were (counter)stained using Nissl staining (cresyl violet acetate solution) for 45 min, dehydrated, and coverslipped. Slides were imaged with a Zeiss Axioskope 2 light microscope equipped with a high-resolution digital camera (C4742-95, 72 px/inch, Hamamatsu Photonics, Italy), using objective lens spanning from 2.5 × to 40 ×.

### Immunofluorescence

Immunofluorescence staining was performed as previously described [14]. Sections were incubated with the primary antibodyfor 48 h at 4 °C. Following washes with PBS, sections were incubated with the appropriate secondary antibody (Alexa Fluor 488 anti-rabbit IgG, Alexa Fluor 546 anti-rabbit IgG; 1:200; Invitrogen, Carlsbad, CA, USA) for 2 h. Sections were mounted and coverslipped using the anti-fade solution Vectashield (Vector Laboratories).

### Western Blotting

Fresh brain tissues (7D tumor *n* = 3; 14D tumor *n* = 3; 21D tumor *n* = 3; SHAM *n* = 4) were mechanically homogenized in NaCl 0.1 M, Tris HCl 0.01 M pH 7.6, EDTA 0.001 M pH 8, and protease inhibitors (PMSF 0.5 mM, Roche; Complete Mini Tablets, Roche). Protein concentration was measured using the Bradford method and the lysates were diluted in Laemmli’s buffer (Tris HCl 0.1 M pH 6.8, SDS 4%, BBF 0.2%, Glycerol 20%, Triton 1%, ddH_2_O) containing 4% β-mercaptoethanol in order to have 25, or 40 μg proteins for each sample, according to the protein concentration recommended for the specific primary antibody. Lysates were denatured at 95 °C for 8 min and loaded on a 1.5 mm SDS polyacrylamide mini gel (8–10–15%). Electrophoresis was performed at 100 V for 120 min. The proteins were transferred overnight to nitrocellulose 0.45 um or polyvinylidene fluoride (PVDF) 0.22 um membranes at 30 V, 4 °C. The success of gel electrophoresis and transfer was verified by staining the membranes with the reversible diazo dye Ponceau S (P3504, Sigma-Aldrich) to reveal protein bands with its red/pink stain while leaving a clear background [15, 16]. After blocking of non-specific sites by 5% milk in 20 mM Tris–HCl pH 7.4, 0.2% Tween 20 (TBST), membranes were incubated overnight with primary antibody. After washing in TBST, membranes were incubated with the appropriate HRP-conjugated secondary antibody (Thermo Fisher Scientific, 1:10,000) in the blocking solution for 60 min at room temperature. The signal was detected by the chemiluminescence reaction (Immobilon Western, HRP Substrate, Merck Millipore) and visualized on X-ray film (Fujifilm). The films were digitalized as TIFF images. The density of each specific band was measured with a computer-assisted imaging analysis system (MCID 7.1; Imaging Res. Inc.). To compare the differences between control and treatment groups, we first normalized the density of each specific band against the density of the corresponding internal loading band. β-Actin or Ponceau S was used to assess equal loading among samples [16]. The Western blotting experiments were repeated at least three times for each marker.

### Primary Antibodies

The following antibodies were used for immunodetection: rabbit antibodies against glial fibrillary acidic protein (GFAP) (1:2500, Sigma-Aldrich, G9269, Saint Louis, USA); rabbit antibodies to ionized calcium binding adaptor molecule 1 (Iba1) (1:500, Wako Chemicals, 019–19,741, USA); rabbit antibodies against Ki67 (1:250, Abcam, ab16667, Cambridge, UK); rabbit antibodies to tenascin-C (1:100, Abcam, ab108930, Cambridge, UK); rabbit antibodies to TMEM119 (1:5000, Proteintech, 66,948–1-Ig, IL, USA); rabbit antibodies to CCL2 (1:500, Novus bio, NBP1-07,035, Milan, Italy); rabbit antibodies against MHC Class II (MHCII) (1:500, Abcam, ab180779, Cambridge, UK); rabbit antibodies to MMP9 (1:500, Invitrogen, JA80-73, Waltham, USA); rabbit antibodies to Fibulin-2 (1:500, Invitrogen, AB_11153545, Waltham, USA); mouse antibodies to β-Actin (1:10,000, Sigma, Saint Louis, USA).

### The UALCAN Database Inquiry

University of ALabama at Birmingham CANcer (UALCAN) database is a portal for gene expression and survival analyses inquiries based on human cancers [17, 18]. Data mining was performed using the following conditions: “Genes: FBLN2, MMP9, CCL2, TMEM119, HLA-DPB1”; “Analysis Type: Cancer (*n* = 156) vs. Normal (*n* = 5)”; “Cancer Type: Glioblastoma multiforme”; “Data Type: TCGA dataset, mRNA expression levels”. The database supported graphical output (boxplot and heatmap) and statistical analysis of data expressed with median and interquartile range (IQR) analyzed with student t-test, considering unequal variance.

### Measurements and Statistical Analysis

Measurements of immunohistochemistry (IHC), immunofluorescence (IF), and western blotting (WB) markers in the right and left brain hemispheres were accomplished using computer-assisted image analysis system (MCID 7.1; Imaging Res. Inc., Canada). All data were collected in a blinded manner; the observer making the measurements was not aware of the group. The data were exported and converted to a density distribution histogram using the Sigma-Plot 10.0 program (SPSS-Erkrath). Data were checked for normal distribution and homogeneity of variance by the Kolmogorov–Smirnov’s and Levene’s mean tests, respectively. Normal distributions with equal variances were analyzed by Student’s *t* tests for comparing two groups, and one-way ANOVA for multiple comparisons followed by all pairwise Holm–Sidak post hoc test. Kruskal–Wallis test was used for non-parametric analysis followed by Tukey’s test for pairwise multiple comparisons. Level of significance was set at *p* = 0.05 (*p ≤ 0.05; **p ≤ 0.01; ***p ≤ 0.001). Results are expressed as group mean ± SEM and represented in vertical bar charts. Individual images were assembled and the same adjustments made for resolution (300 px/inch), brightness, contrast and sharpness using Adobe Photoshop (Adobe Systems).

## Results

### Glioblastoma Time Course

**Fig. 1:**
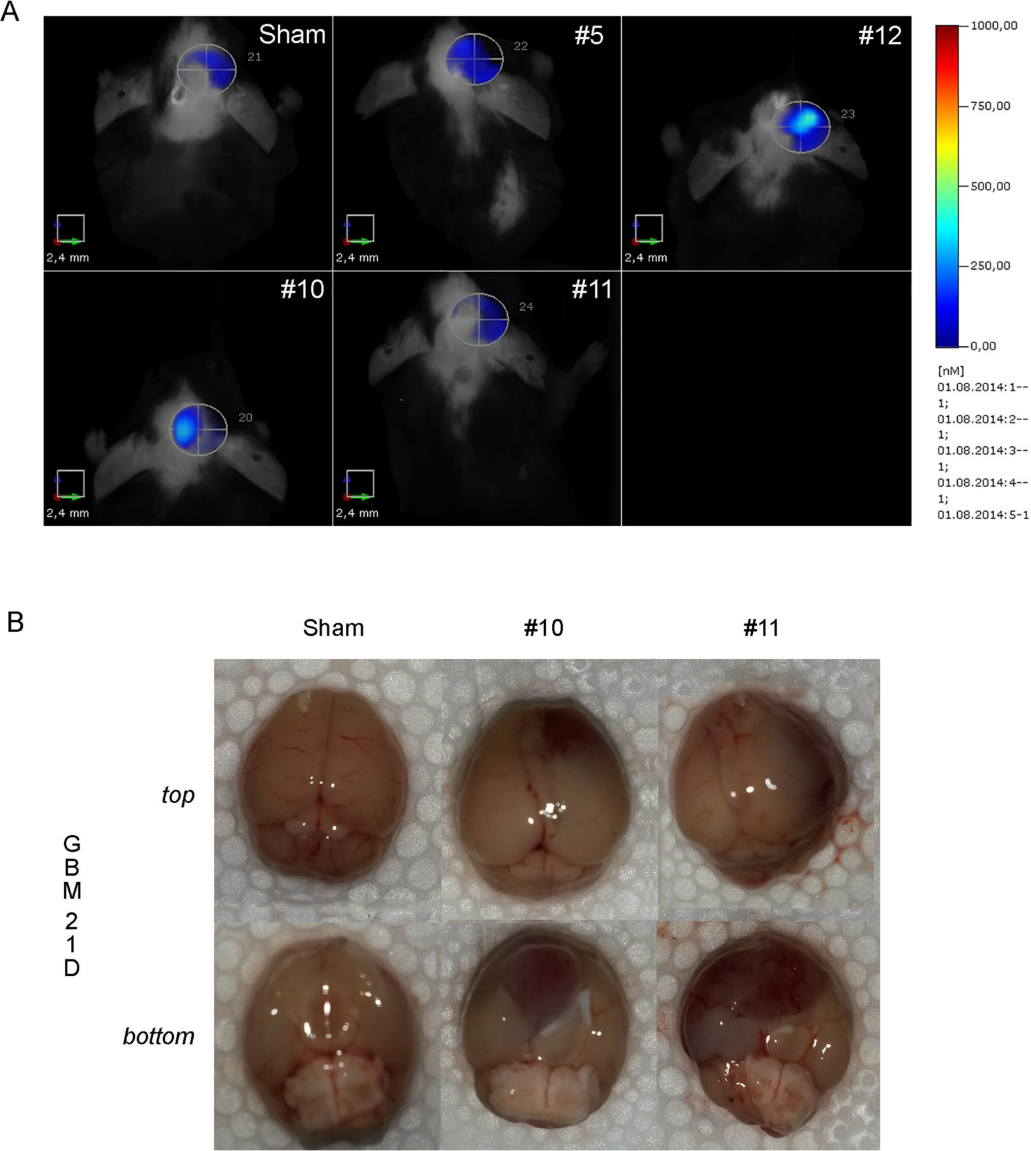
Tumor monitoring at 21D. **A** C57Bl/6 mice with vehicle (SHAM) or tumor cells on day 21 (21D). Mice were intravenously injected with 10 nmol of 2-DG-750 probe or vehicle 750 dye and imaged at 21D FMT 1000 Imaging System (Perkin Elmer). The SHAM mouse showed no specific targeting. Bioluminescence imaging showed. **B** Macroscopic photographs of a Sham and two scanned 21D tumor-bearing brains. The GBM development was differently oriented from the original tumor cells injection site

**Fig. 2:**
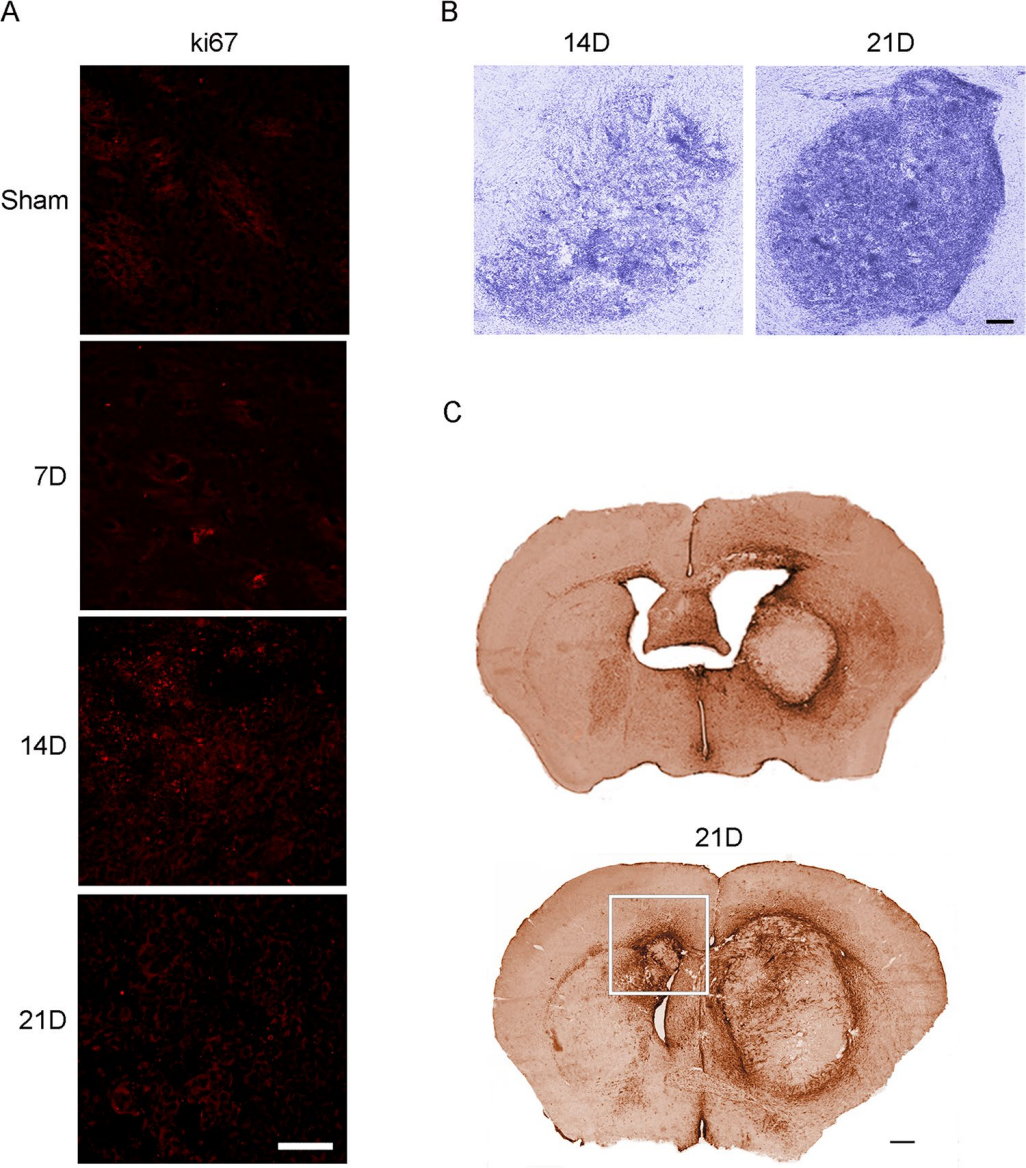
Glioblastoma time-course. **A** ki67 Immunofluorescence in the primary site of injection (right striatum) at different time points. Ki67^+^ cells increase at 14D. Scale bar 20 μm. **B** GBM tumor bulk at 14D and 21D stained by cresyl violet acetate. The tumor tissue appears as necrotic. Massive infiltration occurs at 21D. Scale bar 50 μm. **C** 2D reconstruction of immuno-stained tumor-bearing slices at 14D and 21D for GFAP. GFAP^+^ astrocytes surround the tumor mass. A secondary mass (white square) is evident in the contralateral hemisphere at 21D. Scale bar 100 μm

We performed a time-course study to investigate the modifications of the microenvironment during the progression of the GBM in our immunocompetent mouse model. GL261 tumor-bearing mice have a survival of 26–32 days after cell implantation [19]. Therefore, we considered 21D as terminal stage of GBM progression. Microenvironmental cues act as variables of a complex system to determine the local and distant invasiveness of GBM. As a pilot study, we used the non-invasive 3D fluorescence tomography to verify the success of the tumor development after the injection protocol and to preliminarily evaluate the accuracy of the technique in predicting GBM development in vivo. The 3D fluorescence tomography is based on the elevated glucose uptake due to the tumor's high metabolism compared to the surrounding tissue [20]. We noticed a highly heterogeneous glucose uptake in the acquired area within the 21D tumor-bearing group (Fig. 1A). The GBM metabolism (total pmol 17.717 ± 4.557) had a mean value of 2.15-fold higher than the SHAM (total pmol 8.236). However, we did not perform a quantitative analysis due to the limited numerosity of the sample (Supplementary Fig. 1A, B). Moreover, other than the high heterogeneity of the 21D group, one animal demonstrated lower glucose uptake than the SHAM. At the necropsy analysis, we observed that the tumor masses had successfully developed in all animals. However, the GL261-injected mice showed tumor masses with different positions in the brain (Fig. 1B). Hence, the tumor bulk was not always included in the determined volumetric ROI of the scan. This qualitative analysis supported the scarce reliability of the 3D tomography tool in GBM evaluation in vivo. GL261 cells and their in-vivo development showed several features similar to the human high-grade glioma. Among these, they did not express GFAP [21]. Seven days after the injection of the GL261 cells in the mice striatum, the distribution of the ki67-positive cells appeared spotted. However, the striatum architecture was compromised compared to the SHAM group. The ki67-positive signal at the injection site peaked after 14 days and was quenched on day 21 (Fig. 2A). The histological analysis of the striatum using Nissl staining showed a well-defined tumor bulk at 14D with several necrosis areas (Fig. 2B) and a high cell density in the same area on day 21 (Fig. 2B). Moreover, the GBM invaded the contralateral hemisphere in 75% of 21D animals (Fig. 2C). Altogether, these data suggested that the GBM grows prevalently as a bulky mass during the first 2 weeks after the inoculation of GL261 cells. However, the necrotic, primary GBM tissue may be infiltrated by phagocytic cells in the terminal stage. Contralateral GBM invasion is visible at 21D suggesting the establishment of a secondary mass between the second and third week after tumor cells injection.

### Glioblastoma-Associated Astrocytes

**Fig. 3:**
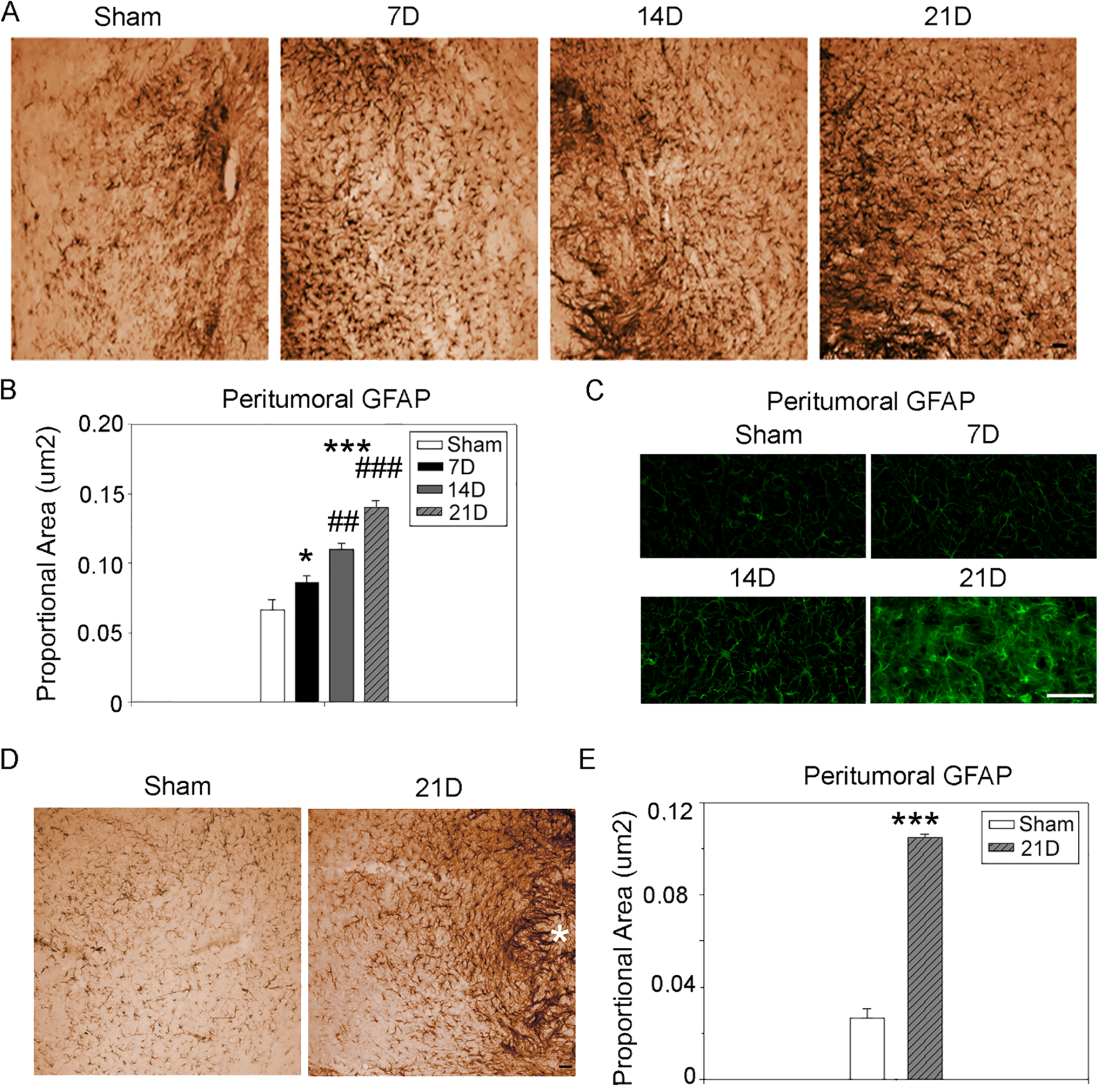
Astrocytes dynamic during GBM progression. **A** GBM-associated astrocytes expressing GFAP increase with GBM progression. Scale bar 50 μm. **B** Quantification of peritumoral astrocytes immunostained for GFAP. The results are expressed as proportional area (One-way Anova for multiple comparisons, post-hoc Holm-Sidak correction, *p ≤ 0.05; **p ≤ 0.01; ***p ≤ 0.001 for comparisons vs. SHAM; ^#^*p* ≤ 0.05; ^##^*p* ≤ 0.01; ^###^*p* ≤ 0.001 for comparisons between the stages of GBM progression). **C** Morphological changes in representative high-magnified GFAP-expressing tumor-associated astrocytes. Scale bar 50 μm. **D** GBM-associated astrocytes expressing GFAP in the contralateral (left) hemisphere under SHAM condtion and at 21D. Scale bar 50 μm. The secondary tumor mass is indicated with an asterisk. **E** Quantification of peritumoral astrocytes immunostained for GFAP in the contralateral hemisphere. The results are expressed as proportional area. (*p ≤ 0.05; **p ≤ 0.01; ***p ≤ 0.001, Student’s *t* test)

Astrocytes are the CNS resident cells that may share a common origin with the GBM cells [2]. We found that astrocytes enwrap the tumor bulk at 14D and 21D forming a barrier-like structure (Fig. 2C). The immunohistochemistry analysis of the peritumoral GFAP expression revealed an increasing intensity of the signal (0.086 ± 0.007 7D, *p* = 0.023; 0.110 ± 0.004 14D *p* ≤ 0.001; 0.140 ± 0.004, *p* ≤ 0.001 21D) compared to both the SHAM group (0.064 ± 0.007) and between the time-points (7D vs. 14D *p* = 0.0038; 14D vs. 21D *p* = 0.0007) (Fig. 3A, B) with a gradient appearance of limiting reactive astrocytes surrounding the injection site. A gradient of morphological modifications such as cell body hypertrophy and terminals arborization occurring during the reactive astrogliosis was also appreciated following the time course (Fig. 3C). The immunohistochemical overexpression of GFAP in the peritumoral contour was evident in the secondary mass (0.105 ± 0.001) compared to the same brain regions of the sham animals (0.0265 ± 0.004, p<0.001) (Fig. 3D, E). These data support a dynamic interplay between GBM and astrocytes that follows the time course of the pathology.

### Extracellular Matrix Remodeling

**Fig. 4:**
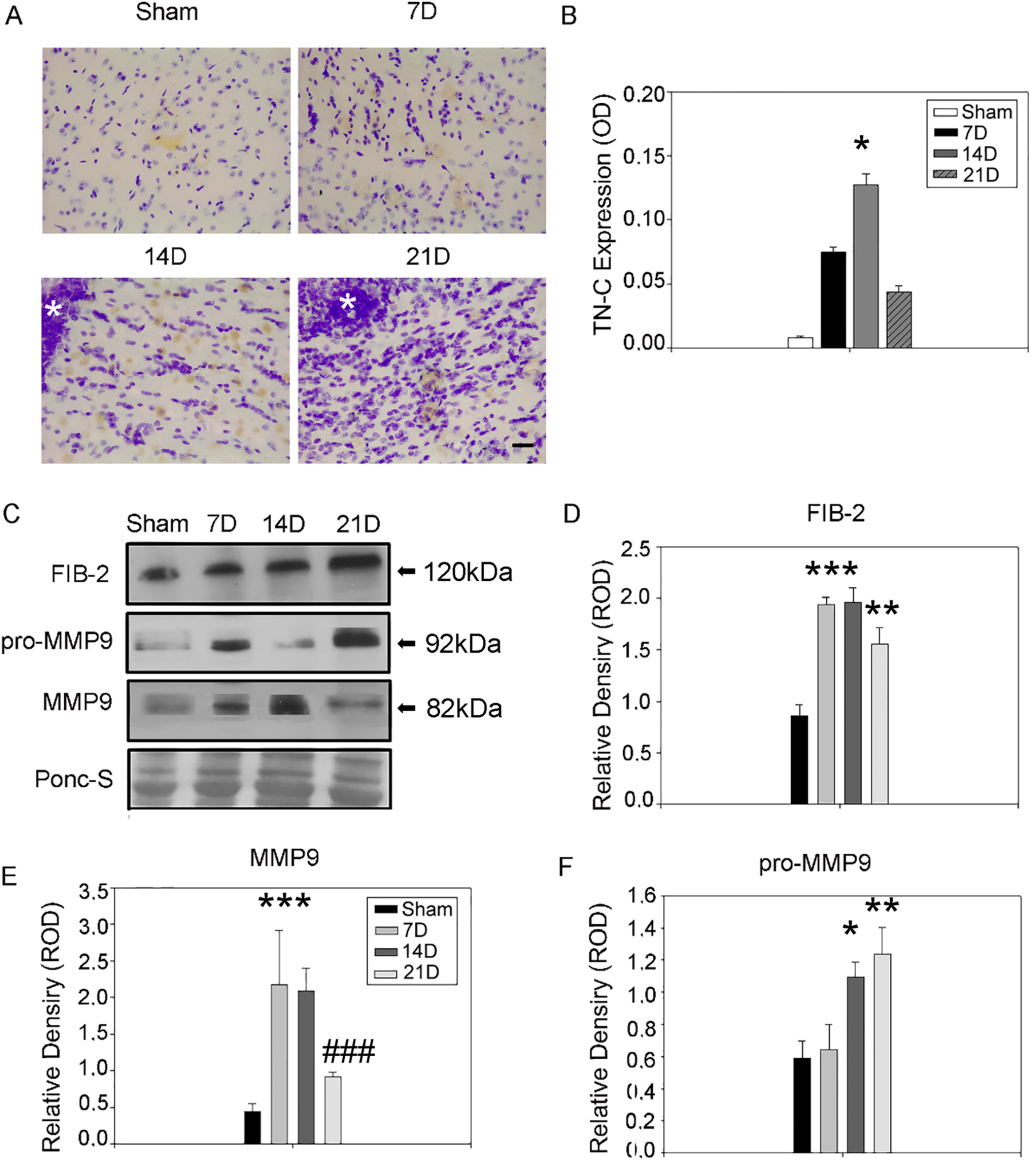
Extra-cellular matrix remodeling during GBM progression. **A** TN-C immunohistochemistry in the peritumoral region. TN-C peak is evident at 14D. Brain sections were counterstained with cresyl violet acetate (Nissl staining). Scale bar 20 μm. **B** Quantification of peritumoral TNC. The results are expressed as a measure of optical density (Kruskal–Wallis test was used for non-parametric analysis followed by Tukey’s test for pairwise multiple comparisons. *p ≤ 0.05; **p ≤ 0.01; ***p ≤ 0.001 for comparisons vs. SHAM; ^#^*p* ≤ 0.05; ^##^*p* ≤ 0.01; ^###^*p* ≤ 0.001 for comparisons between the stages of GBM progression). **C** Western blot of right hemisphere lysate showed differential expression for FIB-2, pro-MMP9, and MMP9 during GBM progression. Samples derive from the same experiment; gels/blots were processed in parallel. **D–F** Quantification of FIB-2, MMP-9, pro-MMP9 western blot bands relative to the total protein content stained with Ponceau-S. FIB-2 and MMP-9 upregulation was prominent in the earlier stages of GBM development. The expression of MMP-9 was in equilibrium with its precursor pro-MMP-9(One-way Anova for multiple comparisons, post-hoc Holm-Sidak corrections. Kruskal–Wallis test was used for non-parametric analysis followed by Tukey’s test for pairwise multiple comparisons. *p ≤ 0.05; **p ≤ 0.01; ***p ≤ 0.001 for comparisons vs SHAM; ^#^*p* ≤ 0.05; ^##^*p* ≤ 0.01; ^###^*p* ≤ 0.001 for comparisons between the stages of GBM progression)

Tumor-associated astrocytes and GBM are the main producers of the TN-C [7, 22]. TN-C is a glycoprotein of the ECM associated to the glioma cells invasion and provides attachment sites to the migrating cells [23]. The immunohistochemical analysis showed a peak of TN-C at 14D in the peritumoral tissue (0.127 ± 0.008) compared to the SHAM (0.002 ± 0.00; *p* = 0.016) (Fig. 4A, B), suggesting the decision *to go* taken by the GBM. Accordingly, a secondary tumor mass located in the contralateral hemisphere was established between the second and the third week after the injection (Fig. 2C).

Fibulin-2 is a structural interlinking protein whose overexpression can supply the loss of TN-C and other ECM proteins [8]. Fibulin-2 seems to be associated with advanced high-grade astrocytomas. However, its role in GBM growth or invasiveness has not been verified [9]. The Western blot analysis revealed the upregulation of fibulin-2 at 7D (1.939 ± 0.066) compared to the SHAM group (0.859 ± 0.109, *p* = 0.0002), maintained during the following time points (14D 1.960 ± 0.141; *p* = 0.0002; 21D 1.556 ± 0.158, *p* = 0.004) (Fig. 4C, D). The Fibulin-2 could be pivotal to preserve the integrity of the parenchyma, being associated with both the growth and invasion of the GBM model.

Both the GBM growth and the invasiveness processes require ECM remodeling, mainly conducted through novel deposition of proteins and the digestion of the existing functional scaffold by proteolytic activation of the pro-metalloproteases (pro-MMPs) to metalloproteases (MMPs). Among these, MMP9 expression is the most relevant for positive correlation with brain cancer progression [24]. The Western blot analysis of the MMP9 expression revealed an early upregulation at 7D (2.917 ± 0.076; *p* < 0.0001) maintained at 14D (2.084 ± 0.318; *p* = 0.001), and reduced after 21D (0.496 ± 0.190; *p* = 0.662) almost to SHAM levels (0.611 ± 0.167) (Fig. 4C–E). The expression of MMP-9 at 21D was also significantly reduced if compared to the previous time-points of the disease (7D vs. 21D *p* = 0.00009; 14D vs. 21D *p* = 0.001) (Fig. 4C–E). The Western blot measurements of pro-MMP9 showed no changes at 7D (0.644 ± 0.158; *p* = 0.779), a moderate expression at 14D (1.096 ± 0.093; *p* = 0.022), and peaked at 21D (1.235 ± 0.170; *p* = 0.006) compared to the SHAM (0.591 ± 0.108) (Fig.. 4C–F). Therefore, these data suggest a dynamic equilibrium between the enzyme and the pro-form. The production of novel pro-MMP9 is balanced by its proteolytic activation, that decrease from the first to the last time point. Altogether, these data indicate that the GBM interacting with the resident brain elements induced a selective reorganization of the ECM *to grow* (MMP-9) and *to go* (MMP-9 and TN-C), maintaining the integrity of the tissue during all phases, through interlinking protein deposition (Fibulin-2).

### Glioblastoma-Associated Microglia and Macrophages Spatiotemporal Features of Immune Response

**Fig. 5:**
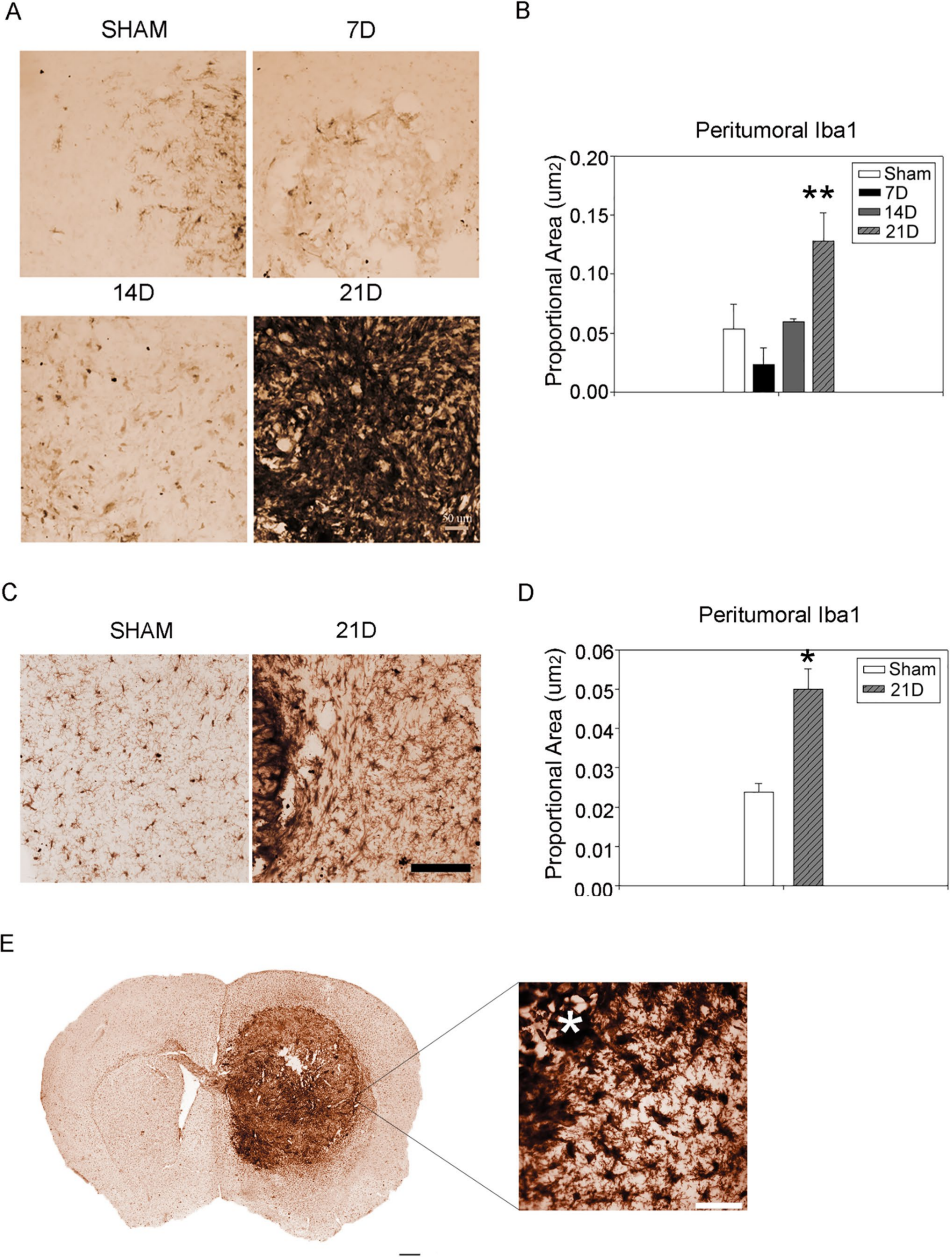
Microglia/macrophages dynamic during GBM progression. **A** Immunohistochemistry for Iba1 showed a bland reaction by microglia/macrophages in the site of the primary GL261 cells injection. A massive infiltration is evident at 21D. Scale bar 50 μm. **B** Quantification of peritumoral Iba1-expressing cells in the peritumoral region, right hemisphere. The results are expressed as proportional area (Kruskal–Wallis test was used for non-parametric analysis followed by Tukey’s test for pairwise multiple comparisons. *p ≤ 0.05; **p ≤ 0.01; ***p ≤ 0.001 for comparisons vs. SHAM; ^#^*p* ≤ 0.05; ^##^*p* ≤ 0.01; ^###^*p* ≤ 0.001 for comparisons between the stages of GBM progression). **C** Immunohistochemistry for Iba1 showed an increase of Iba1^+^ cells at 21D compared to the SHAM group in the contralateral hemisphere. **D** Quantification of peritumoral Iba1-expressing cells in the peritumoral region, contralateral hemisphere. The results are expressed as proportional area (*p ≤ 0.05; **p ≤ 0.01; ***p ≤ 0.001, Student’s *t* test). **E** 2D reconstruction of tumor-bearing section immunostained for Iba1 at 21D. Scale bar 100 μm. The morphology of Iba1 expressing cells at the tumor border is shown in detail. Scale bar 50 μm. The tumor bulk is indicated with an asterisk*

**Fig. 6:**
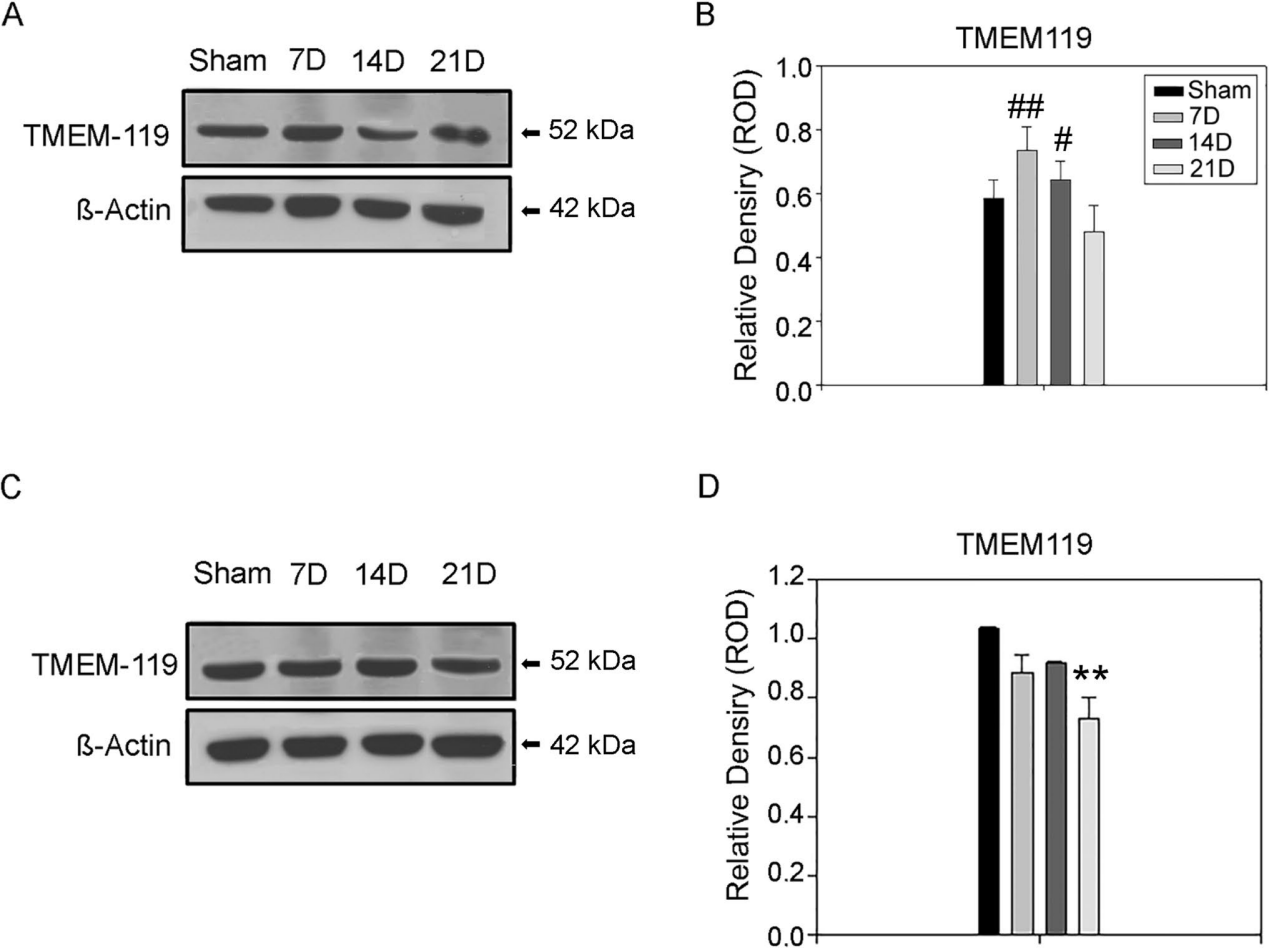
Microglia reaction appears inhibited during GBM progression. **A** Western blot analysis of right hemisphere lysate revealed differential expression for microglia specific marker TMEM119 during GBM progression. Samples derive from the same experiment; gels/blots were processed in parallel. **B** Quantification of TMEM119 Western blot bands relative to the β-actin content used as the internal loading control. The expression of TMEM119 does not differ from the SHAM group at 7D but is downregulated at 21D (One-way Anova for multiple comparisons, post-hoc Holm-Sidak corrections. Kruskal–Wallis test was used for non-parametric analysis followed by Tukey’s test for pairwise multiple comparisons. *p ≤ 0.05; **p ≤ 0.01; ***p ≤ 0.001 for comparisons vs. SHAM; ^#^*p* ≤ 0.05; ^##^*p* ≤ 0.01; ^###^*p* ≤ 0.001 for comparisons between the stages of GBM progression). **C** Western blot of contralateral hemisphere lysate showed differential expression for microglia specific marker TMEM119 during GBM progression. Samples derive from the same experiment; gels/blots were processed in parallel. **D** Quantification of TMEM119 western blot bands relative to the β-actin content from the contralateral hemisphere. The expression of TMEM119 is downregulated at 21D compared to the SHAM group in the contralateral hemisphere (One-way Anova for multiple comparisons, post-hoc Holm-Sidak corrections. Kruskal–Wallis test was used for non-parametric analysis followed by Tukey’s test for pairwise multiple comparisons. *p ≤ 0.05; **p ≤ 0.01; ***p ≤ 0.001 for comparisons vs SHAM; ^#^*p* ≤ 0.05; ^##^*p* ≤ 0.01; ^###^*p* ≤ 0.001 for comparisons between the stages of GBM progression)

Microglia and macrophages are the first-line defense against pathogens and traumatic injuries. However, they are polarized by the GBM, and support its progression [25]. To study the impact of the tumor progression on microglia and macrophages cells, we examined their recruitment and morphological changes through the immunostaining of Iba1, expressed by both the cytotypes. The analysis revealed no significant recruitment of microglia and macrophages in the peritumoral area at 7D (0.011 ± 0.007, *p* = 0.068) and 14D (0.059 ± 0.002, *p* = 0.639) compared to the SHAM group (0.074 ± 0.007). In the last phase, both the primary site of injection and the surrounding tissue were highly infiltrated by Iba^+^ elements (0.132 ± 0.033, *p* = 0.0024) in the homolateral hemisphere (Fig. 5A, B). Moreover, the contralateral hemisphere showed infiltration of Iba1^+^ cells at 21D (0.0500 ± 0.005, *p* = 0.030) compared to the same brain areas in the SHAM group (left, 0.0238 ± 0.002) (Fig. 5C, D). In particular, the corpus callosum and the external capsule were interested by Iba1^+^ cells reaction (Fig. 5E). Iba1^+^ cells appeared to have an ameboid shape at 21D (Fig. 5E). Although activated microglia and macrophages share the morphological features, peripheral-derived-macrophages are abundant in the glioblastoma tissue and have different transcriptional states compared to their brain resident counterparts [26]. Transmembrane protein 119 (TMEM119) is a cell-surface protein and a specific microglial marker, and is not expressed by macrophages or other immune or neural cell types [27]. The Western blot analysis of TMEM119 bands from the right hemisphere revealed a non significant increasing trend at 7D (0.735 ± 0.072, *p* = 0.117) and 14D (0.644 ± 0.056, *p* = 0.514) compared to the SHAM group. However, at 21D the TMEM119 expression was significantly lower (0.427 ± 0.066) compared to the previous stages (7D, *p* = 0.009; 14D, *p* = 0.044) (Fig. 6A, B). Matching results were obtained with the analysis of the TMEM119 protein expression in the contralateral hemisphere. No changes were found at 7D (0.882 ± 0.060, *p* = 0.0431) and 14D (0.920 ± 0.002, *p* = 0.0998) compared to the SHAM group (1.031 ± 0.0074). However, there was a significant down expression of TMEM119 at 21D (0.729 ± 0.073, *p* = 0.002) (Fig. 6C, D). These findings suggest an overall inhibition of resident microglia with tardive macrophagic invasion of the parenchyma, both in the primary mass and following the migratory routes (e.g., white matter tracts).

**Fig. 7:**
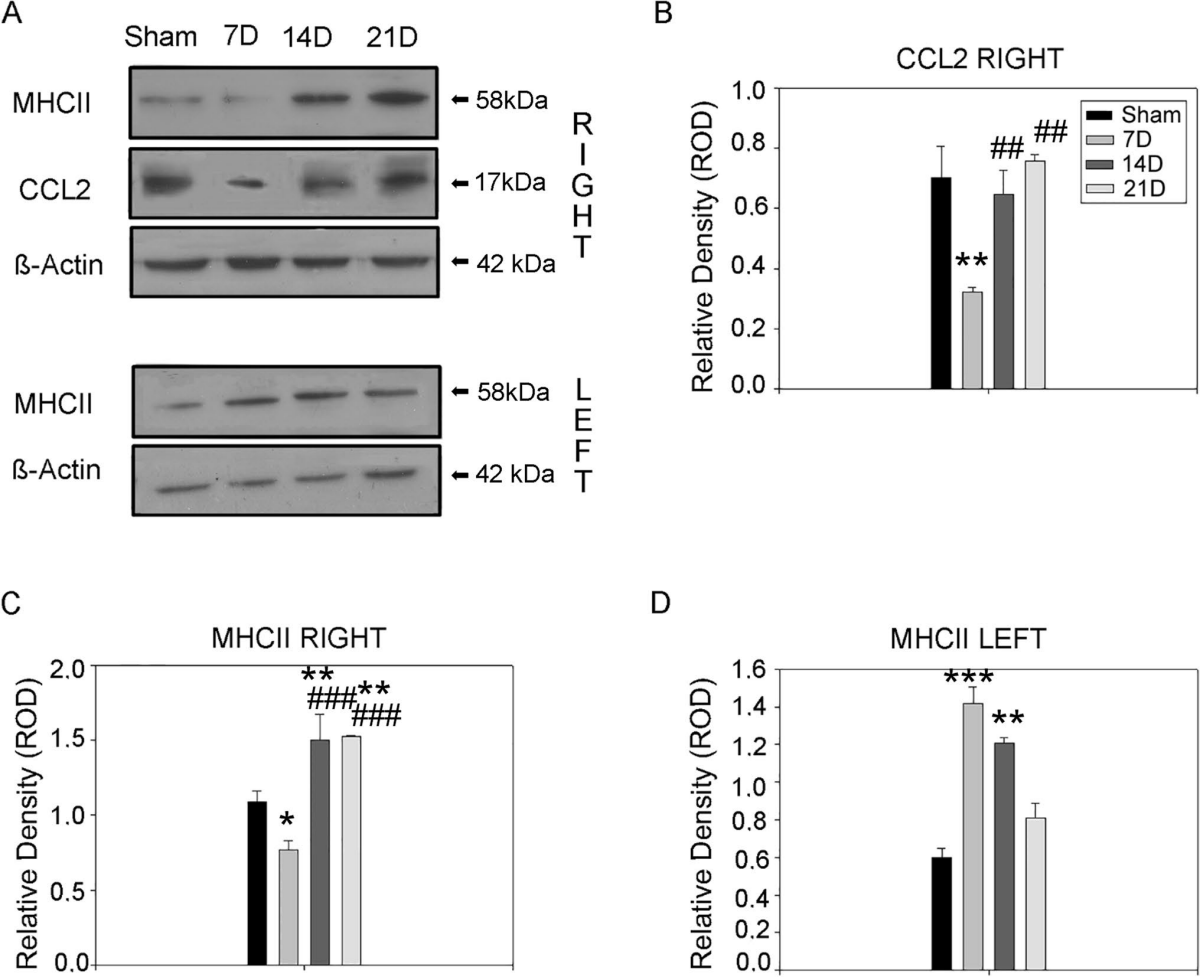
Antigen-presenting function and microglia/macrophage-associated inflammation are compromised in the initiation of the GBM. **A** Western blot analysis of right (top) and left (bottom) hemisphere lysate revealed differential expression for MHCII and CCL2 during GBM progression. Samples derive from the same experiment; gels/blots were processed in parallel. **B** Quantification of CCL2 right hemisphere western blot bands relative to the β-actin content used as the internal loading control (One-way Anova for multiple comparisons, post-hoc Holm-Sidak corrections. Kruskal–Wallis test was used for non-parametric analysis followed by Tukey’s test for pairwise multiple comparisons. *p ≤ 0.05; **p ≤ 0.01; ***p ≤ 0.001 for comparisons vs SHAM; ^#^*p* ≤ 0.05; ^##^*p* ≤ 0.01; ^###^*p* ≤ 0.001 for comparisons between the stages of GBM progression). **C**, **D** Quantification of MHCII western blot bands relative to the β-actin content used as internal loading control from the right and left hemisphere, respectively (One-way Anova for multiple comparisons, post-hoc Holm-Sidak corrections. Kruskal–Wallis test was used for non-parametric analysis followed by Tukey’s test for pairwise multiple comparisons. *p ≤ 0.05; **p ≤ 0.01; ***p ≤ 0.001 for comparisons vs SHAM; ^#^*p* ≤ 0.05; ^##^*p* ≤ 0.01; ^###^*p* ≤ 0.001 for comparisons between the stages of GBM progression)

Monocyte chemoattractant protein-1 (MCP-1/CCL2) is renowned for driving the chemotaxis of myeloid-derived cells and it is released by the GBM and the activated microglia and macrophages [28, 29]. The analysis of the western blot bands demonstrated CCL2 reduced expression at 7D (0.323 ± 0.016, *p* = 0.0037) compared to the SHAM group (0.703 ± 0.103). However, the protein level increased at 14D (0.646 ± 0.080, *p* = 0.008) and 21D (0.759 ± 0.019, *p* = 0.0016) compared to the early phases of the pathology (Fig. 7A, B).

Activated microglia and macrophages are the main cell type in the brain that present pathological antigens to the adaptive immune system (i.e., lymphocytes) via the molecular histocompatibility complex I and II. Considering that GL261 cells do not express the major histocompatibility complex II (MHCII) molecules [21], we used this marker to study the functional response of the microglia and macrophages during the GBM experimental time course. The western blot analysis revealed lower levels of MHCII at 7D (0.773 ± 0.058, *p* = 0.023) compared to the SHAM group (1.091 ± 0.073) with a stable increment at 14D (1.498 ± 0.173, *p* = 0.014) and 21D (1.524 ± 0.007, *p* = 0.010) (Fig. 6A–C). The increment of MHCII expression was significantly different as compared to the previous stages of the disease (7D vs. 14D *p* = 0.0008; 7D vs. 21D *p* = 0.0007). Taken together, these data suggest that functional response related to the antigen presentation and myeloid cells recruitment is a tardive phenomenon in the GBM time course. Moreover, resident microglia appears inhibited, particularly in the early stages.

To investigate the regional differences of immune activation, we compared MHCII and CCL2 expression levels in the left hemispheres (contralateral to the site of injection). MHCII Western blot of the left hemispheres showed higher levels in the experimental groups at 7D (1.420 ± 0.087, *p* = 0.0014) and 14D (1.206 ± 0.301, *p* = 0.0094) compared to the SHAM (0.597 ± 0.051) with a reduction at 21D (0.811 ± 0.077, *p* = 0.287) (Fig. 7A–D). CCL2 measurements showed similar but not significant trends of expression (data not shown). The regional expression of these markers is related to the GBM challenge that may hamper the antigen-presenting properties of myeloid cells.

### Ualcan Database Analysis

The gene transcription expression was summarized in a heatmap generated by the database routine (Supplementary Fig. 2A). The Fibulin-2 transcriptional expression was found downregulated in human glioblastoma compared to normal samples (16.17 IQR 9.09; 1.64 IQR 2.99; *p* = 0.0045), apparently in contrast with our data (Supplementary Fig. 2A-B). However, we traced a reduction of fibulin-2 at the last time point (21D). Human data do not follow the GBM time course but are more representative of this final stage. MMP9 mRNA data expression from the UALCAN database was in accordance with our results, being the tumor group median value (17.63 IQR 35.06) higher compared to the normal group (0.25 IQR 0.33, *p* < 0.0001; Supplementary Fig. 2A-C). The concordance between transcription in humans and the protein expression in the murine model further confirm the involvement of MMP9 in all phases of GBM development, growth, and dissemination.

The expression data of CCL2 mRNA in human GBM (113.98 IQR 164.12) relative to the healthy population (67.86 IQR 48.32; *p* = 0.71) were similar to the mouse protein expression found in our GBM model at 21D (Supplementary Fig. 2A-D). MHCII expression levels were considered with the study of the HLA-DPB1 gene, which was found upregulated in tumors (224.82 IQR 181.89) compared to healthy tissue (62.53 IQR 32.67), thus matching the protein concentration in the final stage of our model (Supplementary Fig. 2A–E). The expression of the specific microglial marker TMEM119 (Supplementary Fig. 2A–F) was upregulated in the human GBM (20.59 IQR 23.767) compared to the healthy population (15.803 IQR 8.156; *p* = 0.0079). However, we did not find relevant changes in the protein expression at the late stage compared to the SHAM in our model, but an increase at early stage. Supposedly the involvement of microglia, as a time-related event, may differ between humans and mice.

## Discussion

TME is a fundamental regulator of cancer progression and heterogeneity modified by aging, endogenous and exogenous stressors, with a central role of the inflammatory cascade [30]. GBM microenvironment includes brain resident cells, ECM, and vascular components. In the present study, we analyzed the temporal modifications of the TME using a syngeneic mouse model. Immunocompetent mouse models develop a tumor resembling human GBM and are the most appropriate to study the immune challenge of TME and the tumor-stroma interactions [31]. Consistent with other reports, GL261 tumor infiltrates the normal tissue and peaks in proliferation by 2 weeks post-inoculation, resulting in an evident mass. However, we found an heterogeneous detection of the glucose consumption in the in-vivo fluorescence scanning in the last phase of the disease. The mean glucose uptake measurement was higher than the SHAM level (Supplementary Fig. 1), but lower than the typical enhancement expected for a tumor [20]. This result may account for the heterogeneity of bulk formation and invasion of the tumor cells, which reinforces the notion of dynamic interactions between the tumor and the microenvironment. However, we need to assess the limitation of the volumetric scan that allows for deep tridimensional acquisition within a fixed ROI that could not visualize the tumor metabolism coherently in each scanned animal. Within the mature primary tumor core (21D), central necrotic regions are evident [32]. According to both human and mouse studies [33], 75% of the animals had a secondary tumor mass in the contralateral hemisphere by 21D, suggesting the tumor invasiveness prevails in the third-week post-inoculation (Fig. 2). Several factors including cytokines and ECM regulate the glioma invasiveness. The upregulation of the large ECM glycoprotein TN-C is associated with the GBM cells migration [7]. High levels of the interleukin IL-33 could potentially elicit TN-C overexpression in TME via NfkB [34]. TN-C cleavage by MMPs leads to the exposure of an adherent site that can spread the tumor cells migration in a positive feedback loop [23]. Accordingly, we found the TN-C peak at 14D (Fig. 4), confirming the high invasiveness of our model at this stage. In contrast, GBM local growth mechanisms prevailed in the early pathogenesis. Fibulin-2 overexpression was found as compensatory mechanism for the loss of TN-C, TN-R, neurocan, and brevican along with fibulin-1, and allowed the maintenance of a normal brain structure [8]. The measured changes of TN-C expression did not affect fibulin-2 levels, that are maintained constitutively high in this pathological model. Fibulin-2 has an emergent role in carcinogenesis and high-grade gliomas [35] and has been recently proposed as a prognostic biomarker for meningiomas [36]. However, while we observed an increase of fibulin-2 expression in GL261 tumor-bearing mice as compared to sham, fibulin-2 transcriptional expression is frequently downregulated in human glioblastoma (Supplementary Fig. 2). Although the discrepancy between the observed fibulin-2 overexpression in our model and the expression levels in human gliomas warrants for further investigation, it is interesting to note that we observed a reduction of fibulin-2 protein expression at 21D time points as compared to 7D and 14D, and this finding is consistent with fibulin-2 reduced protein levels in high-grade gliomas as compared to low-grade gliomas in humans [9]. To the best of our knowledge, this is the first report of a fibulin-2 overexpression in a GBM mouse model.

Both the GBM growth and invasiveness require a re-organization of the tissue accomplished by the activity of the MMPs. Among MMPs, MMP9 has been associated with the glioma grade and its upregulation to a poor prognosis [37]. MMP9 expression is not specific of a single-cell population, being secreted in the ECM by both tumor and healthy cells [38–40]. We found the upregulation of MMP9 at both 7D and 14D, confirming the MMP9 involvement in local growth and dissemination of GBM respectively. Moreover, our findings were consistent with the expression levels in human GBM as compared to normal tissue (Supplementary Fig. 2).

Activated astrocytes and microglia/macrophages showed different reactions to the developing tumor. Astrocytes surrounded the tumor since the early phase and increased in density over time in the peritumoral region (Fig. 3). The local recruitment of reactive astrocytes was visualized through the microscopic imaging of GFAP, which did not co-localize with active proliferating cells marked with ki67 (data not shown). GFAP reaction often correlates to the severity of the injury [41]. The astrocytic peritumoral localization may be due to the presence in the TME of chondroitin sulfate glycosaminoglycans (CS-GAG) that are repellant for astrocytic migration [42]. Astrocytes adjacent to the tumor border appeared elongated in morphology similar to scar-forming astrocytes at focal injuries. Astrocytes beyond this area were hypertrophic and showed gradient-like morphology with normalization of their features as the increasing distance from the tumor core (Fig. 3). Despite the morphological spectrum, the molecular profile of peritumoral astrocytes has been demonstrated to be variable, with many dysfunctional cells subsets [43]. In a previous publication, we found the specificity of p65BTK expression in gemistocytes, an astrocytic subpopulation negatively correlated to human gliomas [44]. However, JAK-STAT activation seems to play a key role in driving the reactive state of tumor-associated astrocytes and in the crosstalk with the microglial/myeloid cells to provide an anti-inflammatory and an immune-suppressive microenvironment [45]. Microglia and macrophages account for over 30–50% of the tumor in the mature GBM and are fundamental for GBM maintenance [46]. As the tumor bulk formed (14D), Iba1^+^ cells were found in the peritumoral region, while they infiltrated the tumor core at 21D (Fig. 5). Surprisingly, we noticed a low presence of Iba1^+^ elements at the injection site in the early phases of GBM development. Many attempts were conducted to distinguish the resident microglia from the monocytes/macrophages, and the findings are discussed controversially [46–48]. GBM cells are the main producers of CCL2 and an increase of CCL2 expression is associated with major infiltration by monocytes in tumor bearing-mice [49]. Moreover, GBM is a highly vascularized solid tumor and its growth induces blood–brain barrier (BBB) pathological breakdown [50]. The scanty recruitment of myeloid cells in the early GBM development (7D) was confirmed by the low expression of CCL2. On the other hand, the expression of CCL2 increased in the late stages to the levels observed in SHAM animals (similarly to what happens in human GBM, Supplementary Fig. 2) along with Iba1. The specific microglial marker TMEM119 was upregulated as protein levels in the earlier stages of the disease in our model and was restored to the SHAM level at the later stage. However, the RNA expression of TMEM119 is found significantly higher than in normal tissues in human GBM (Supplementary Fig. 2), suggesting a different regulation of the microglial response in mice as compared to humans. Overall our results suggest the major contribution of monocytes/macrophages during the third week of GBM experimental development. The same evidence was obtained using GFP^+^-bone-marrow chimeras generated by head-protection irradiation [51]. Moreover, transcriptomics analysis revealed that GBM selectively modified the expression of microglia sensing genes to hamper the tumor-killing capabilities of this cytotype [25]. To analyze the innate immune system function, we considered the protein level of MHCII, which is not expressed by GL261 cells [21]. Besides the lack of recruitment, the downregulation of MHCII indicated a mechanism of immune escape of the tumor. MHCII was upregulated in the region distant to the injection (i.e., contralateral hemisphere) during the early phases of the time course, indicating an inflammatory-like state of the CNS. However, the presence of GBM cells somehow suppressed the expression of this protein in the nearby tissue. The high expression of MHCII in the contralateral hemisphere at the same time point has been reported in a previous study [52]. In contrast, MHCII levels in the right hemisphere increased during the late stages of GBM progression, indicating the potential restoration of the antigen presenting function, as found in human GBM (Supplementary Fig. 2). Our data are in accordance with a study demonstrating the RNA overexpression of MHCII and the costimulatory molecules CD80/CD86 required for the proper function in the GL261-wt mouse model [53]. Despite the recovery of antigen processing and presenting functions by the innate immune system, a low infiltration of the adaptive immune system was found both in the homolateral and in the contralateral hemispheres [53, 52], suggesting other immune regulators are involved in GBM immune escape in the later phases [54]. Interestingly, CCL2 and MHCII expression in the left hemisphere showed a reduction trend at 21D compared to the earlier phases, which could be justified by the secondary tumor mass occurrence.

## Conclusions

In summary, we demonstrated that the GBM-induced microenvironment interferences are complex and dynamic. We showed that microglia, macrophages, and astrocytes, as well as TN-C, MMP-9, and fibulin-2, are differentially regulated with the disease progression. In particular, the microglia/macrophagic response is hindered during the early phase of tumorigenesis and bulk formation, suggesting the existence of a nurturing cradle for GBM cells, that may involve reactive astrocytes. This hypothesis may open a time window for potential time-dependent, targeted therapies.

## Supplementary Information

ESM 1 (PNG 345 kb)

High resolution image (TIF 6968 kb)

ESM 2 (PNG 164 kb)

High resolution image (TIF 6968 kb)

## Data Availability

The datasets generated and/or analyzed during the current study are available from the corresponding author on reasonable request.

## Abbreviations

GBM: Glioblastoma
MMPs: Metalloproteases
ECM: Extracellular matrix
TME: Tumor microenvironment
CNS: Central nervous system
2-DG: 2-Deoxy-d-glucose
IHC: Immunohistochemistry
IF: Immunofluorescence
WB: Western blotting
GFAP: Glial fibrillary acidic protein
Iba1: Ionized calcium binding adaptor molecule 1
TMEM119: Transmembrane protein 119
MHCII: Major histocompatibility complex class II
CCL2: Chemokine (C–C motif) ligand 2
TN-C: Tenascin C
TN-R: Tenascin R
BBB: Blood–brain barrier

## References

[1] Davis M (2016). Glioblastoma: overview of disease and treatment. CJON.

[2] Zong H, Parada LF, Baker SJ (2015). Cell of origin for malignant gliomas and its implication in therapeutic development. Cold Spring Harb Perspect Biol.

[3] Virtuoso A, Giovannoni R, De Luca C (2021). The glioblastoma microenvironment: morphology, metabolism, and molecular signature of glial dynamics to discover metabolic rewiring sequence. IJMS.

[4] Eder K, Kalman B (2015). The Dynamics of interactions among immune and glioblastoma cells. Neuromol Med.

[5] Platten M, Kretz A, Naumann U (2003). Monocyte chemoattractant protein-1 increases microglial infiltration and aggressiveness of gliomas. Ann Neurol.

[6] Zhang J, Sarkar S, Cua R (2012). A dialog between glioma and microglia that promotes tumor invasiveness through the CCL2/CCR2/interleukin-6 axis. Carcinogenesis.

[7] Xia S, Lal B, Tung B (2016). Tumor microenvironment tenascin-C promotes glioblastoma invasion and negatively regulates tumor proliferation. Neuro Oncol.

[8] Rauch U, Zhou X-H, Roos G (2005). Extracellular matrix alterations in brains lacking four of its components. Biochem Biophys Res Commun.

[9] Ren T, Lin S, Wang Z, Shang A (2016). Differential proteomics analysis of low- and high-grade of astrocytoma using iTRAQ quantification. OTT.

[10] De Luca C, Virtuoso A, Papa M, et al (2022) Regional development of glioblastoma: the anatomical conundrum of cancer biology and its surgical implication. Cells 11:.10.3390/cells11081349 10.3390/cells11081349 PMC9025763 35456027

[11] Virtuoso A, Colangelo AM, Maggio N, et al (2021) The spatiotemporal coupling: regional energy failure and aberrant proteins in neurodegenerative diseases. Int J Mol Sci 22:.10.3390/ijms222111304 10.3390/ijms222111304 PMC8583302 34768733

[12] Koks CAE, De Vleeschouwer S, Graf N, Van Gool SW (2015). Immune suppression during oncolytic virotherapy for high-grade glioma; Yes or No?. J Cancer.

[13] Maes W, Rosas GG, Verbinnen B (2009). DC vaccination with anti-CD25 treatment leads to long-term immunity against experimental glioma. Neuro Oncol.

[14] Papa M, Canitano A, Boscia F (2003). Differential expression of the Na+-Ca2+ exchanger transcripts and proteins in rat brain regions. J Comp Neurol.

[15] Sander H, Wallace S, Plouse R (2019). Ponceau S waste: Ponceau S staining for total protein normalization. Anal Biochem.

[16] Romero-Calvo I, Ocón B, Martínez-Moya P (2010). Reversible Ponceau staining as a loading control alternative to actin in Western blots. Anal Biochem.

[17] Chandrashekar DS, Bashel B, Balasubramanya SAH (2017). UALCAN: a portal for facilitating tumor subgroup gene expression and survival analyses. Neoplasia.

[18] Chandrashekar DS, Karthikeyan SK, Korla PK (2022). UALCAN: An update to the integrated cancer data analysis platform. Neoplasia (New York, NY).

[19] Candolfi M, Curtin JF, Nichols WS (2007). Intracranial glioblastoma models in preclinical neuro-oncology: neuropathological characterization and tumor progression. J Neurooncol.

[20] Martelli C, Dico AL, Diceglie C (2016). Optical imaging probes in oncology. Oncotarget.

[21] Szatmari T, Lumniczky K, Desaknai S (2006). Detailed characterization of the mouse glioma 261 tumor model for experimental glioblastoma therapy. Cancer Sci.

[22] Katz AM, Amankulor NM, Pitter K (2012). Astrocyte-specific expression patterns associated with the PDGF-induced glioma microenvironment. PLoS ONE.

[23] Midwood KS, Orend G (2009). The role of tenascin-C in tissue injury and tumorigenesis. J Cell Commun Signal.

[24] Li Q, Chen B, Cai J (2016). Comparative analysis of matrix metalloproteinase family members reveals that MMP9 predicts survival and response to temozolomide in patients with primary glioblastoma. PLoS ONE.

[25] Maas SLN, Abels ER, Van De Haar LL (2020). Glioblastoma hijacks microglial gene expression to support tumor growth. J Neuroinflammation.

[26] Bowman RL, Klemm F, Akkari L (2016). Macrophage ontogeny underlies differences in tumor-specific education in brain malignancies. Cell Rep.

[27] Bennett ML, Bennett FC, Liddelow SA (2016). New tools for studying microglia in the mouse and human CNS. Proc Natl Acad Sci USA.

[28] Fang K-M, Wang Y-L, Huang M-C (2011). Expression of macrophage inflammatory protein-1α and monocyte chemoattractant protein-1 in glioma-infiltrating microglia: involvement of ATP and P2X7 receptor. J Neurosci Res.

[29] Roesch S, Rapp C, Dettling S, Herold-Mende C (2018). When immune cells turn bad—tumor-associated microglia/macrophages in glioma. IJMS.

[30] Ravi VM, Will P, Kueckelhaus J et al (2021) Spatiotemporal heterogeneity of glioblastoma is dictated by microenvironmental interference. Cancer Biol

[31] Lenting K, Verhaak R, ter Laan M (2017). Glioma: experimental models and reality. Acta Neuropathol.

[32] McKelvey KJ, Hudson AL, Prasanna Kumar R (2020). Temporal and spatial modulation of the tumor and systemic immune response in the murine Gl261 glioma model. PLoS ONE.

[33] Mughal AA, Zhang L, Fayzullin A (2018). Patterns of invasive growth in malignant gliomas—the hippocampus emerges as an invasion-spared brain region. Neoplasia.

[34] Zhang J, Tao T, Wang K (2019). IL-33/ST2 axis promotes glioblastoma cell invasion by accumulating tenascin-C. Sci Rep.

[35] Zhang H, Hui D, Fu X (2020) Roles of Fibulin-2 in carcinogenesis. Med Sci Monit 26:. 10.12659/MSM.918099 10.12659/MSM.918099 PMC6977632 31915327

[36] Sofela AA, Hilton DA, Ammoun S (2021). Fibulin-2: a novel biomarker for differentiating grade II from grade I meningiomas. IJMS.

[37] Zhou W, Yu X, Sun S (2019). Increased expression of MMP-2 and MMP-9 indicates poor prognosis in glioma recurrence. Biomed Pharmacother.

[38] Wang M, Wang T, Liu S (2003). The expression of matrix metalloproteinase-2 and-9 in human gliomas of different pathological grades. Brain Tumor Pathol.

[39] Colangelo NW, Azzam EI (2020). Extracellular vesicles originating from glioblastoma cells increase metalloproteinase release by astrocytes: the role of CD147 (EMMPRIN) and ionizing radiation. Cell Commun Signal.

[40] Jiguet-Jiglaire C, Boissonneau S, Denicolai E (2022). Plasmatic MMP9 released from tumor-infiltrating neutrophils is predictive for bevacizumab efficacy in glioblastoma patients: an AVAglio ancillary study. acta neuropathol commun.

[41] Escartin C, Galea E, Lakatos A (2021). Reactive astrocyte nomenclature, definitions, and future directions. Nat Neurosci.

[42] Silver DJ, Siebzehnrubl FA, Schildts MJ (2013). Chondroitin sulfate proteoglycans potently inhibit invasion and serve as a central organizer of the brain tumor microenvironment. J Neurosci.

[43] Campbell SC, Muñoz-Ballester C, Chaunsali L (2020). Potassium and glutamate transport is impaired in scar-forming tumor-associated astrocytes. Neurochem Int.

[44] Sala L, Cirillo G, Riva G (2019). Specific expression of a New Bruton tyrosine kinase isoform (p65BTK) in the glioblastoma gemistocytic histotype. Front Mol Neurosci.

[45] Henrik Heiland D, Ravi VM, Behringer SP (2019). Tumor-associated reactive astrocytes aid the evolution of immunosuppressive environment in glioblastoma. Nat Commun.

[46] Chen Z, Feng X, Herting CJ (2017). Cellular and molecular identity of tumor-associated macrophages in glioblastoma. Cancer Res.

[47] Glass R, Synowitz M (2014). CNS macrophages and peripheral myeloid cells in brain tumours. Acta Neuropathol.

[48] Yu K, Youshani AS, Wilkinson FL (2019). A nonmyeloablative chimeric mouse model accurately defines microglia and macrophage contribution in glioma. Neuropathol Appl Neurobiol.

[49] Feng X, Szulzewsky F, Yerevanian A (2015). Loss of CX3CR1 increases accumulation of inflammatory monocytes and promotes gliomagenesis. Oncotarget.

[50] Wen L, Peng Y, Wang K (2022). Regulation of pathological BBB restoration via nanostructured ROS-responsive glycolipid-like copolymer entrapping siVEGF for glioblastoma targeted therapeutics. Nano Res.

[51] Müller A, Brandenburg S, Turkowski K (2015). Resident microglia, and not peripheral macrophages, are the main source of brain tumor mononuclear cells: microglia or macrophages in GBM. Int J Cancer.

[52] Crommentuijn MHW, Schetters STT, Dusoswa SA (2020). Immune involvement of the contralateral hemisphere in a glioblastoma mouse model. J Immunother Cancer.

[53] Turkowski K, Brandenburg S, Mueller A (2018). VEGF as a modulator of the innate immune response in glioblastoma. Glia.

[54] Goswami S, Walle T, Cornish AE (2020). Immune profiling of human tumors identifies CD73 as a combinatorial target in glioblastoma. Nat Med.

