# Supplementary figures and images for: Tumor Microenvironment and Immune Escape in the Time Course of Glioblastoma
Source: Mol Neurobiol. 2022 Sep 1;59(11):6857–73. doi: 10.1007/s12035-022-02996-z (PMC9525332; doi:10.1007/s12035-022-02996-z)

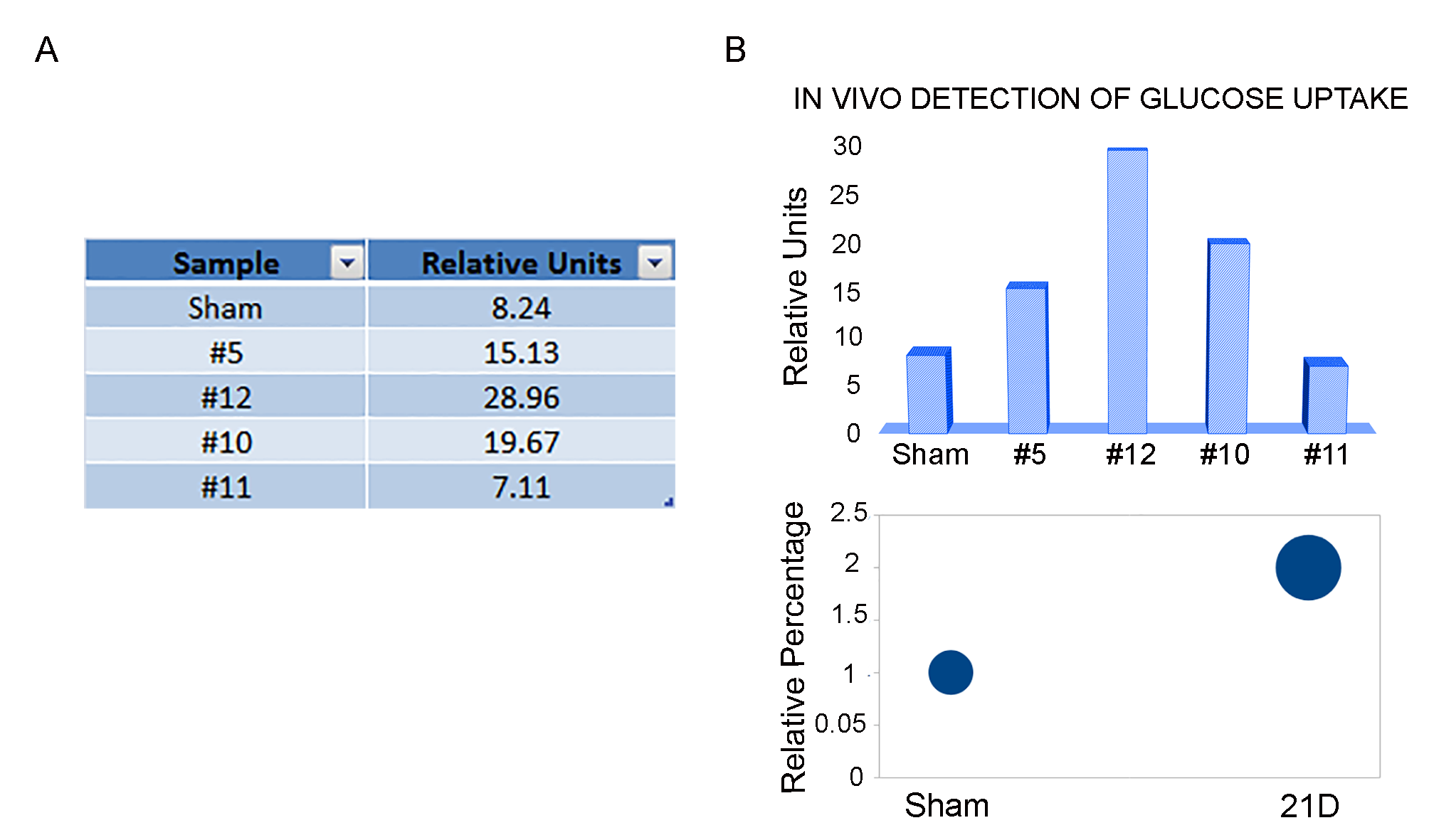

Supplement: Supplementary file 1 — (PNG 345 kb) [file 12035_2022_2996_Fig8_ESM.png]

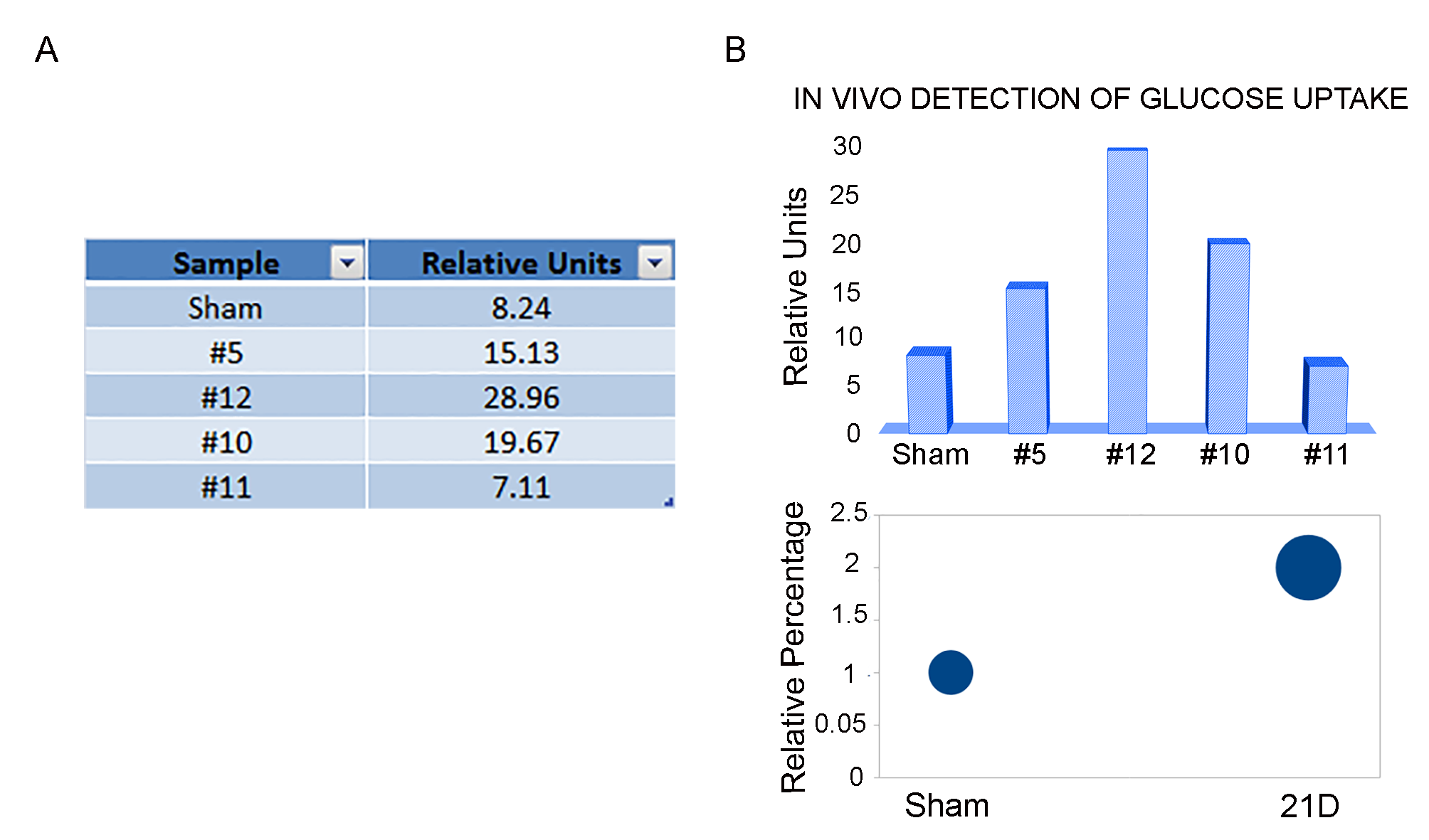

Supplement: Supplementary file 2 — High resolution image (TIF 6968 kb) [file 12035_2022_2996_MOESM1_ESM.tif]

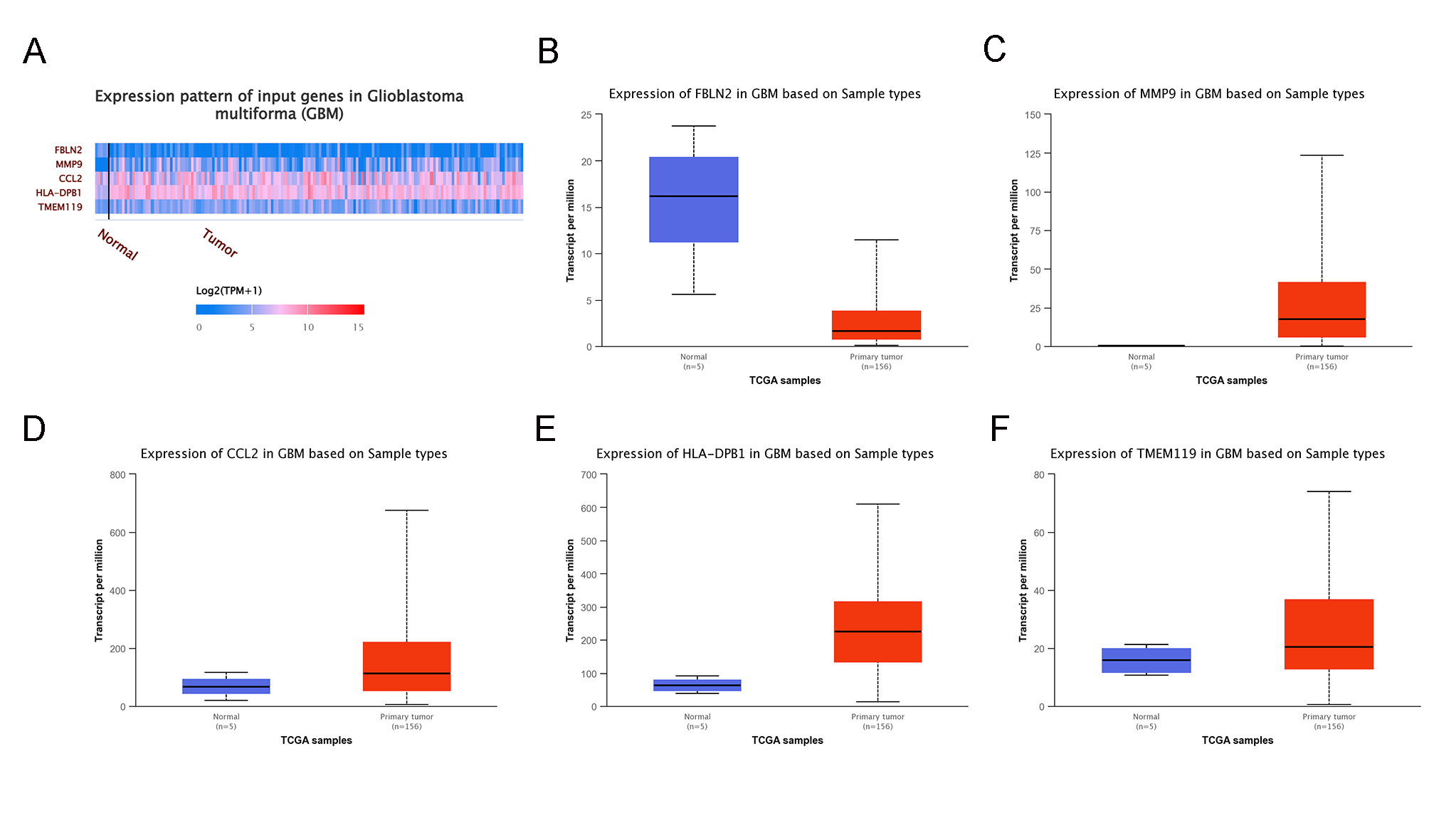

Supplement: Supplementary file 3 — (PNG 164 kb) [file 12035_2022_2996_Fig9_ESM.png]

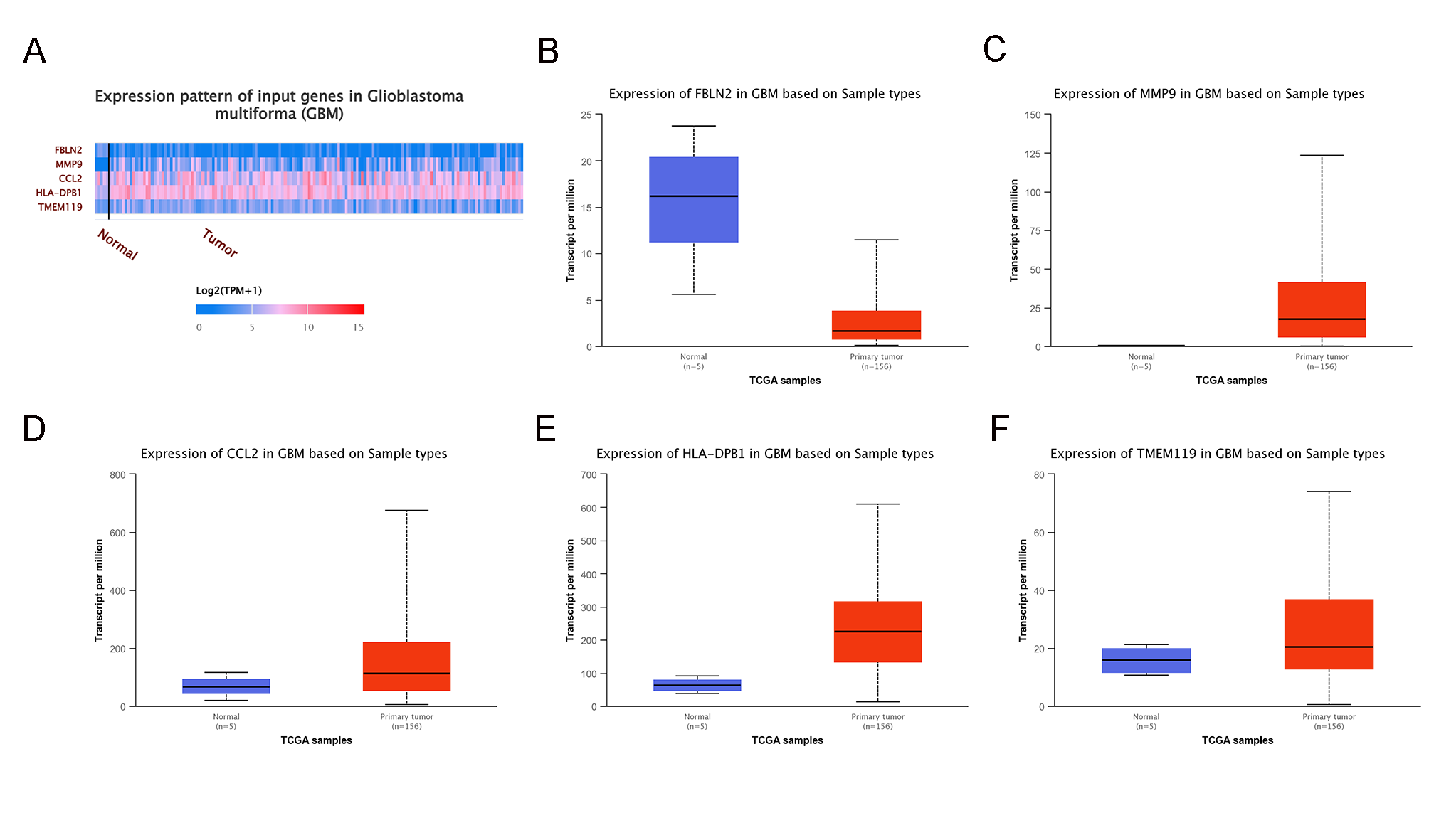

Supplement: Supplementary file 4 — High resolution image (TIF 6968 kb) [file 12035_2022_2996_MOESM2_ESM.tif]
